# Supplementary material for: Therapeutic antisense oligonucleotide mitigates retinal dysfunction in a pig model of CLN3 Batten disease
Source: Nucleic Acids Res. 2025 Nov 4;53(20):gkaf1141. doi: 10.1093/nar/gkaf1141 (PMC12585909; doi:10.1093/nar/gkaf1141)
Supplement: gkaf1141_Supplemental_File [file gkaf1141_supplemental_file.pdf]

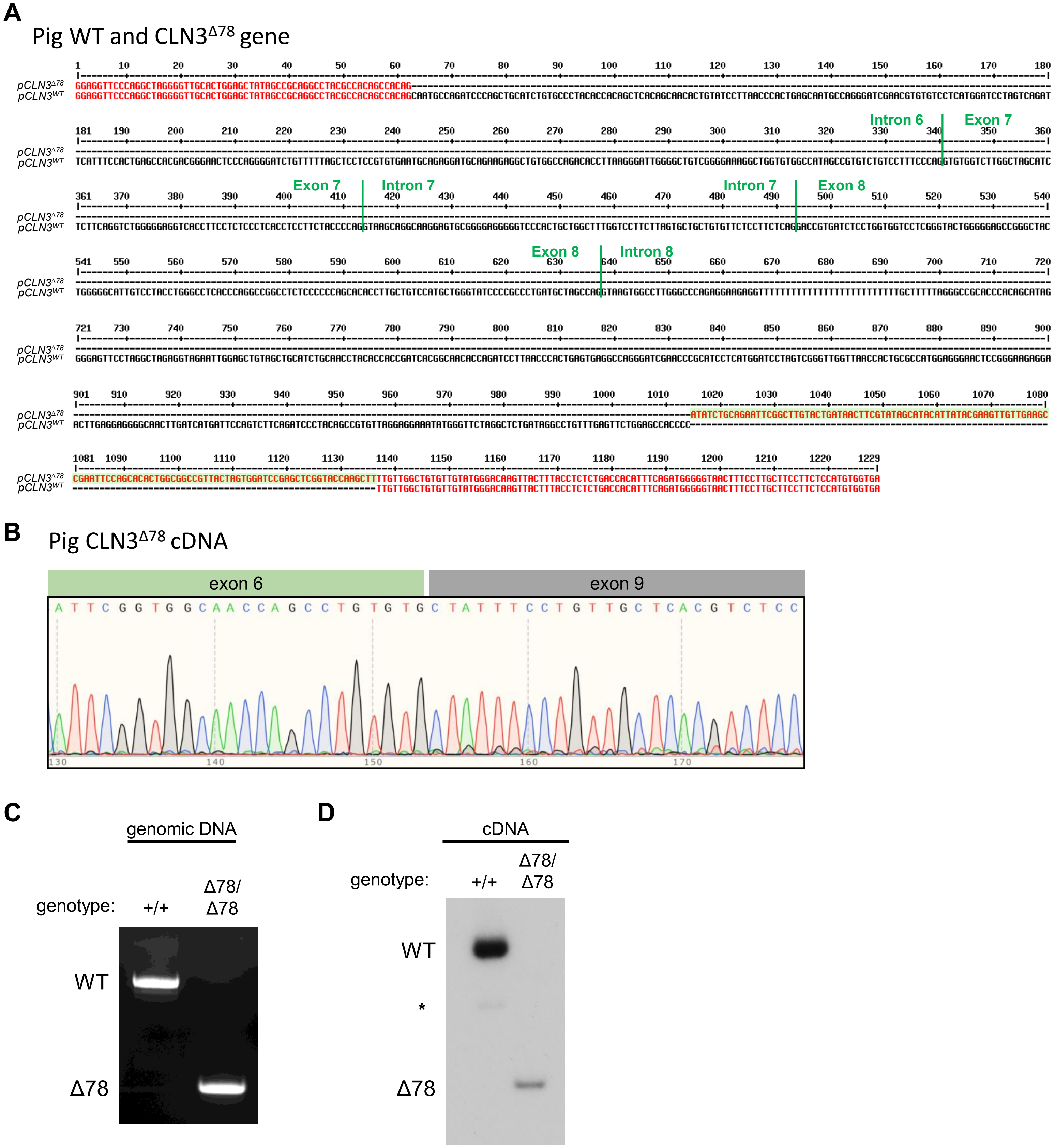

Figure S1. Molecular characterization of the *CLN3*^{Δ78} minipig. (**A**) Sanger sequencing of genomic DNA (gDNA) from a *CLN3*^{Δ78} minipig aligned with *Sus scrofa* whole genome shotgun sequence (AEMK02000016.1), illustrating the borders of the 952 bp deletion. Highlighted in green, the rAAV-mediated gene targeting generation of the *pCLN3*^{Δ78} pig also resulted in a 122bp insertion from the PGK-NeoR cassette used in the process. (**B**) Sanger sequencing chromatogram of cDNA from a *CLN3*^{Δ78} pig confirming splicing of exon 6 to exon 9. (**C**) PCR amplification of genomic DNA from a wild-type (+/+) or homozygote *CLN3*^{Δ78} pig showing exon 7 & 8 deletion. (**D**) Radiolabeled RT–PCR products amplified from cDNA made from RNA isolated from retina of pig with indicated genotypes. Products are labeled on the left side of the gels. **CLN3*^{Δ9}.

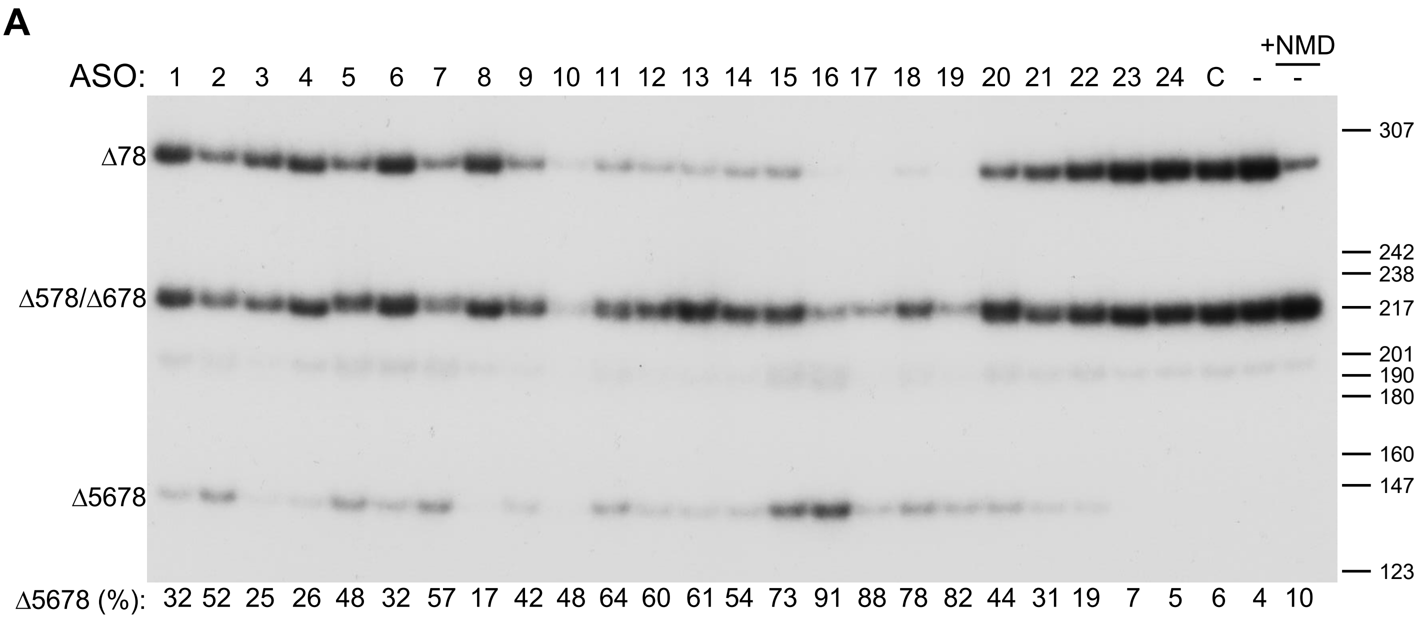

Figure S2. ASOs induce exon 5 skipping to produce *$CLN3^{\Delta578}$* and *$CLN3^{\Delta5678}$* RNA in *$CLN3^{\Delta78}$* pig fibroblasts. (**A**) Radioactive RT–PCR was performed on the cDNA used in Figure 1B from RNA extracted from *$CLN3^{\Delta78}$* cells individually transfected with the indicated ASO, mock treated (C), or untreated (-). PCR was performed using primers in *CLN3* exons 4 and 10. The spliced products resulting from the amplification are labeled on the left of the gel. Below: quantification of exon 5 splicing (calculated as a percentage *$CLN3^{\Delta5678}$*/(*$CLN3^{\Delta78}$*+*$CLN3^{\Delta5678}$*)x100) from a single experiment shown in the gel. Cells were treated with puromycin prior to collection to block nonsense mediated decay (NMD) with the exception of cells from sample labeled “+NMD”. Size markers (bp) are shown on the right of the gel.

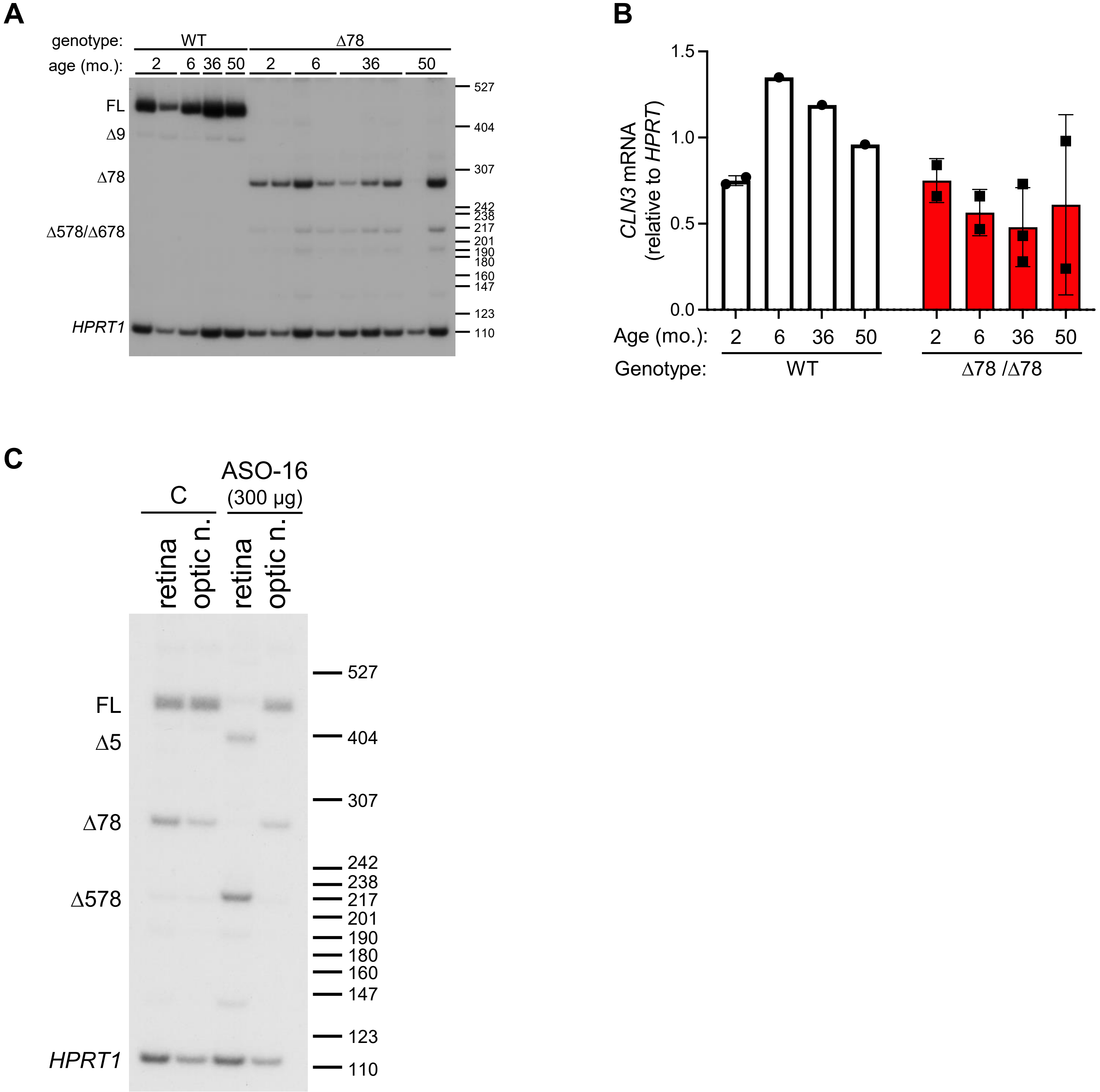

Figure S3. *CLN3* mRNA in the pig retina at different ages. (**A**) Radioactive RT–PCR analysis of *CLN3* in the retina of *CLN3*$^{+/+}$ and *CLN3*$^{\Delta 78}$ pigs at 2, 6, 36, and 50 months (mo.) of age. *HPRT1* was included as a loading control. Products are labeled on the left of the gel. Size markers (bp) are shown on the right of gel. (**B**) Quantification of groups shown in (**A**) with all labeled mRNA isoforms included in quantitation. (**C**) RT-PCR analysis of RNA isolated from the retina and optic nerve tissue of a heterozygote *CLN3*$^{+/\Delta 78}$ pig three months after intravitreal injection ASO-16 (300 μg) into one eye and vehicle (saline, "C") in the contralateral eye. The bands corresponding to each expected splice product are labeled. *HPRT1* was analyzed as a control.

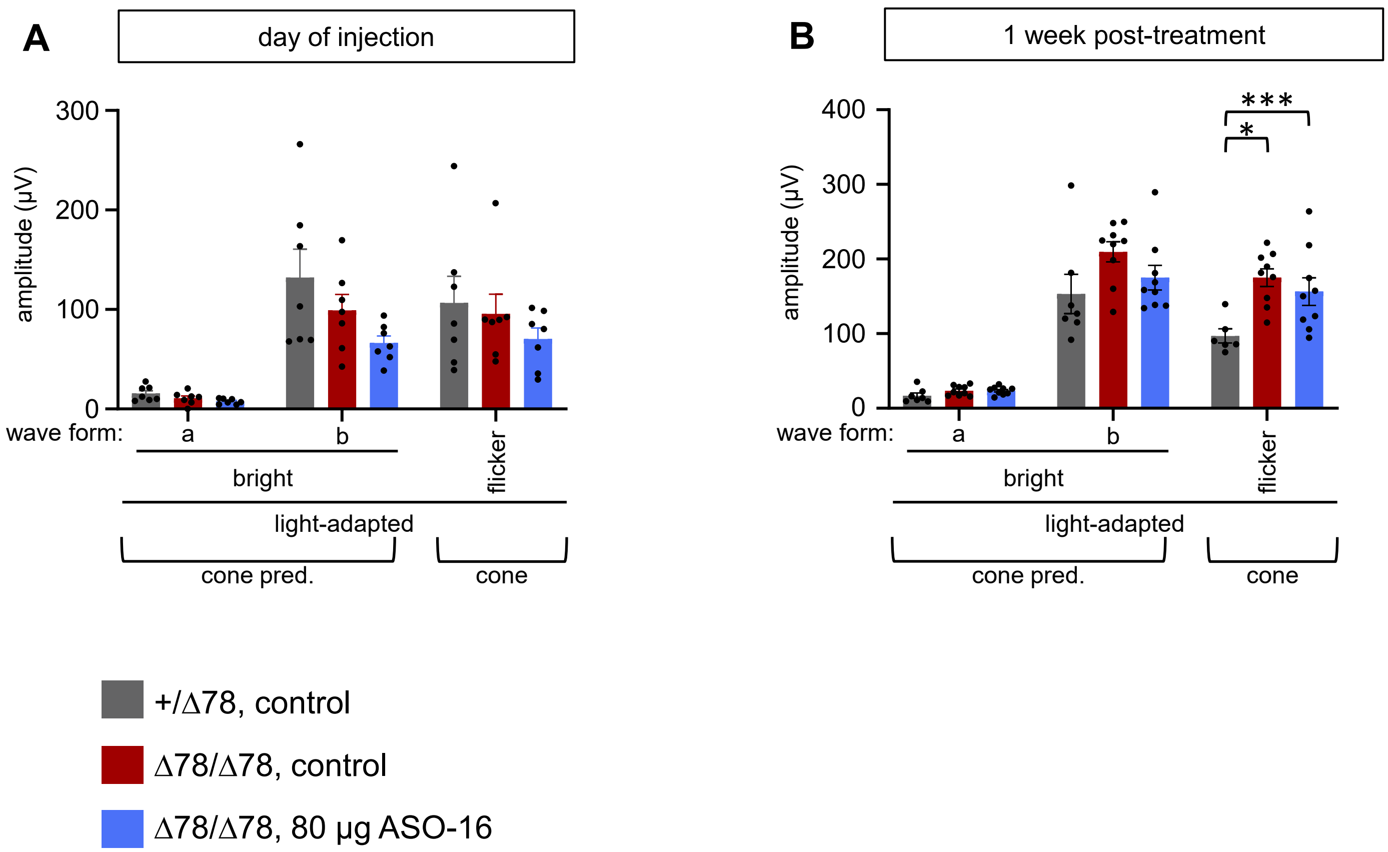

Figure S4. ASO-16 is well-tolerated at 80 μg in the retina of *CLN3*$^{\Delta 78}$ minipigs. ERG raw amplitudes of a-wave, b-wave, and 28.3 Hz flicker at (**A**) on the day of and (**B**) 1 week post injection of DPBS or with 80 μg of ASO-16. Bars show s.e.m.; one-way ANOVA with Dunnett's multiple comparisons; **P* <0.05, ***P* <0.01.

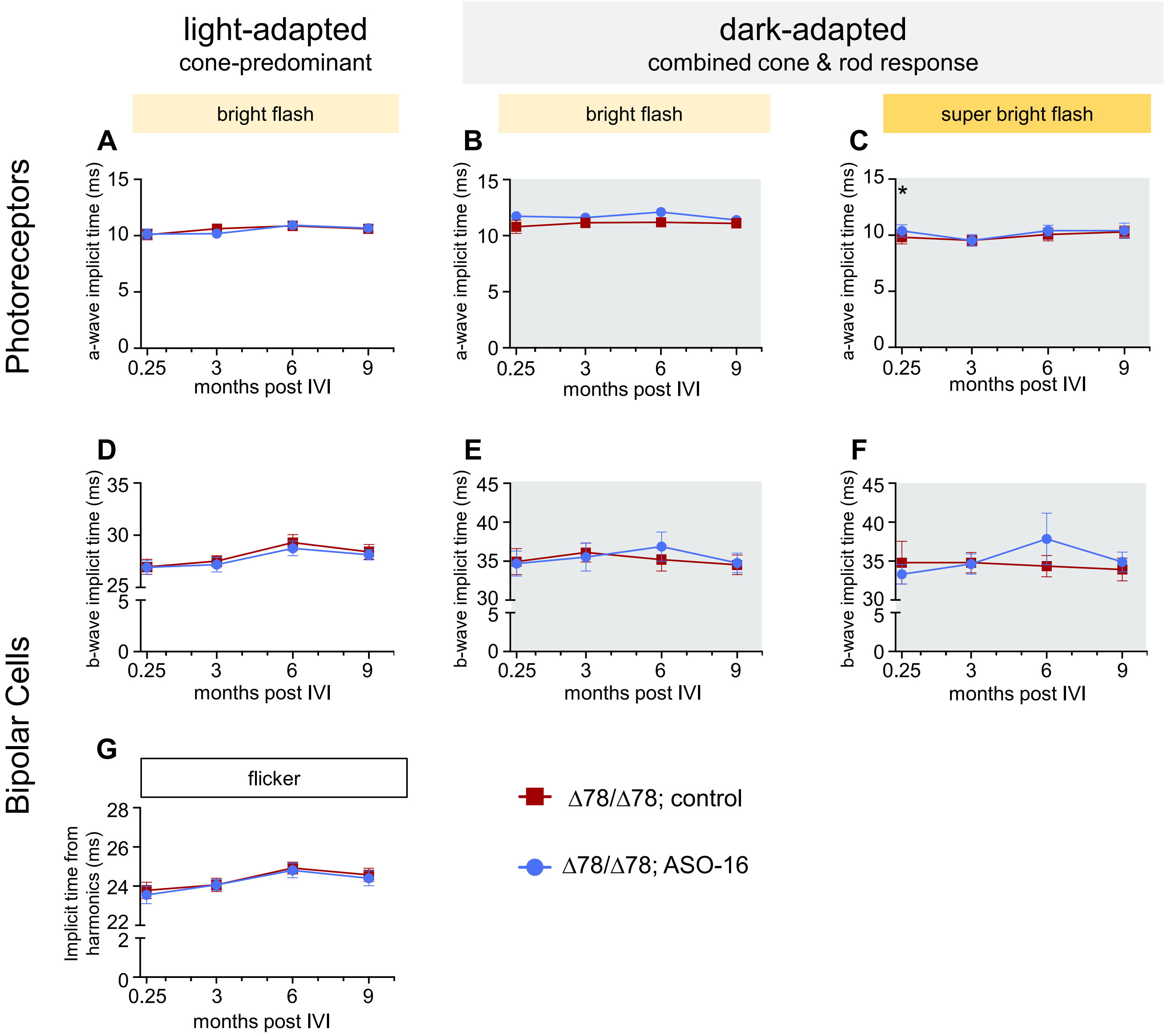

Figure S5. No changes in retinal conduction rate observed in *CLN3*^{Δ78} minipigs. The change in ERG (**A-C**) a-wave implicit time, (**D-F**) b-wave implicit time, and (**G**) implicit time from harmonics is visualized comparing the ASO-treated and vehicle-treated eye of *CLN3*^{Δ78} pigs over time. Bars show s.e.m.; one-tailed paired t-tests; **P* <0.05.

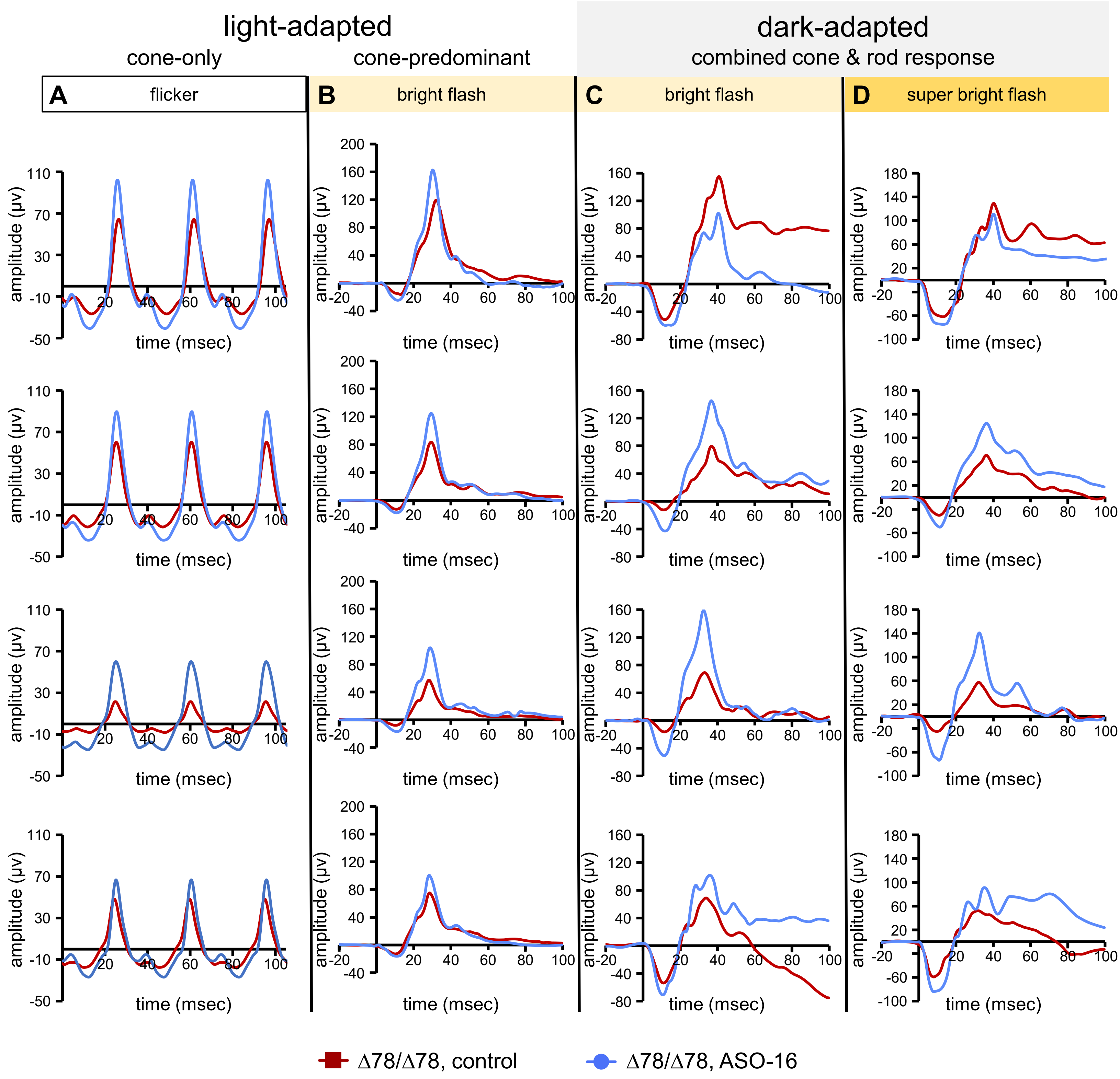

Figure S6. High responder pig ERG waveforms. (**A-D**) Comparison of ERG waveforms from the ASO-treated and vehicle-treated eye of four *CLN3*$^{\Delta 78}$ pigs identified as high responders across the (**A**) 28.3 Hz flicker (8.0 cd•s/m^2), (**B**) light-adapted bright flash (8.0 cd•s/m^2), (**C**) dark-adapted bright flash (8.0 cd•s/m^2), (**D**) or dark-adapted super bright flash (25.0 cd•s/m^2) 9 months post-treatment.

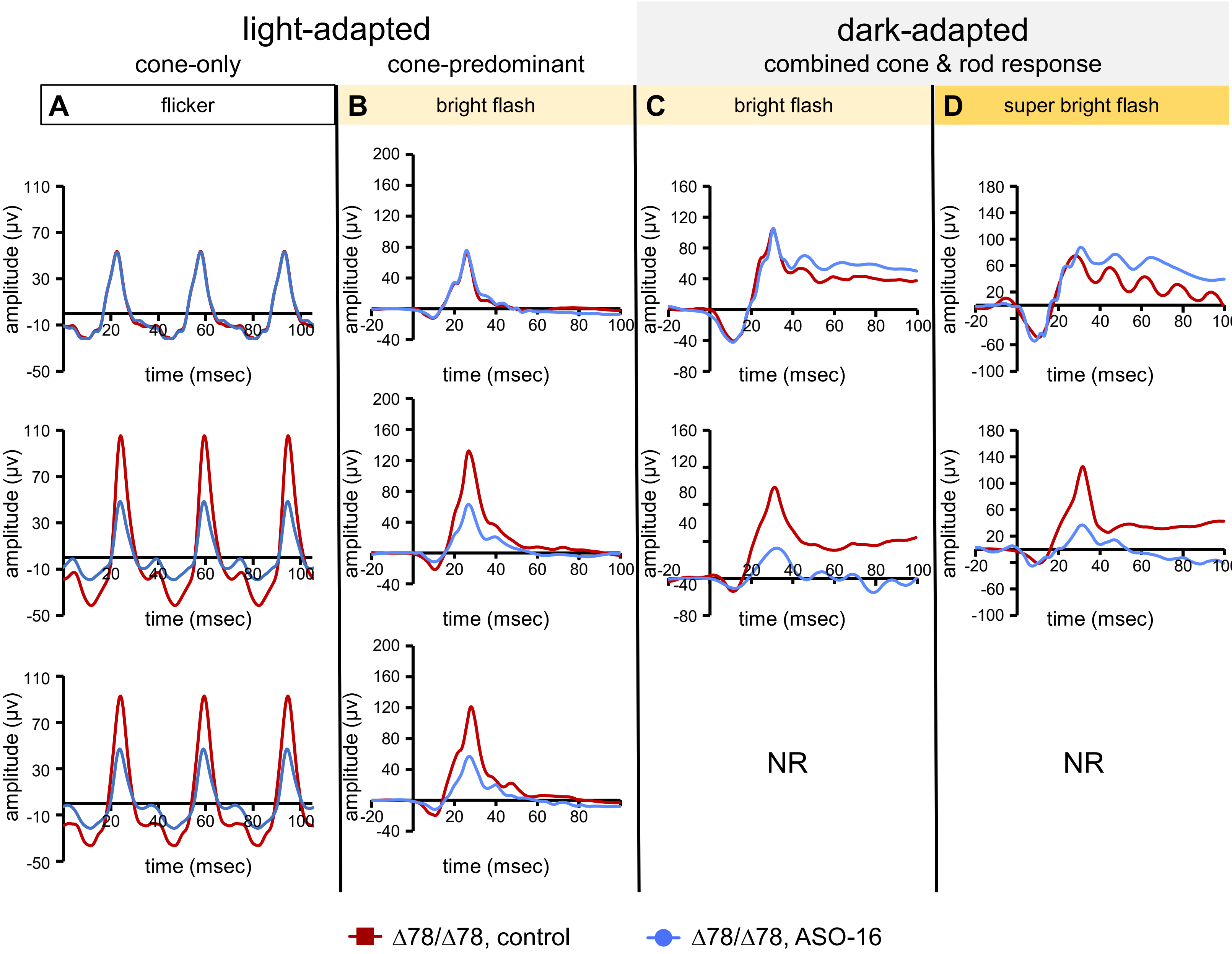

Figure S7. Non-responder ERG waveforms. (**A-D**) Comparison of ERG waveforms from the ASO-treated (blue) and vehicle-treated (red) eye of *CLN3*$^{\Delta 78}$ pigs who did not show improvement in retinal function across the (**A**) 28.3 Hz flicker (8.0 cd•s/m^2), (**B**) light-adapted bright flash (8.0 cd•s/m^2), (**C**) dark-adapted bright flash (8.0 cd•s/m^2), (**D**) or dark-adapted super bright flash (25.0 cd•s/m^2) 9 months post-treatment. (NR) Poor recordings were omitted due to background noise making the waveforms unreliable.

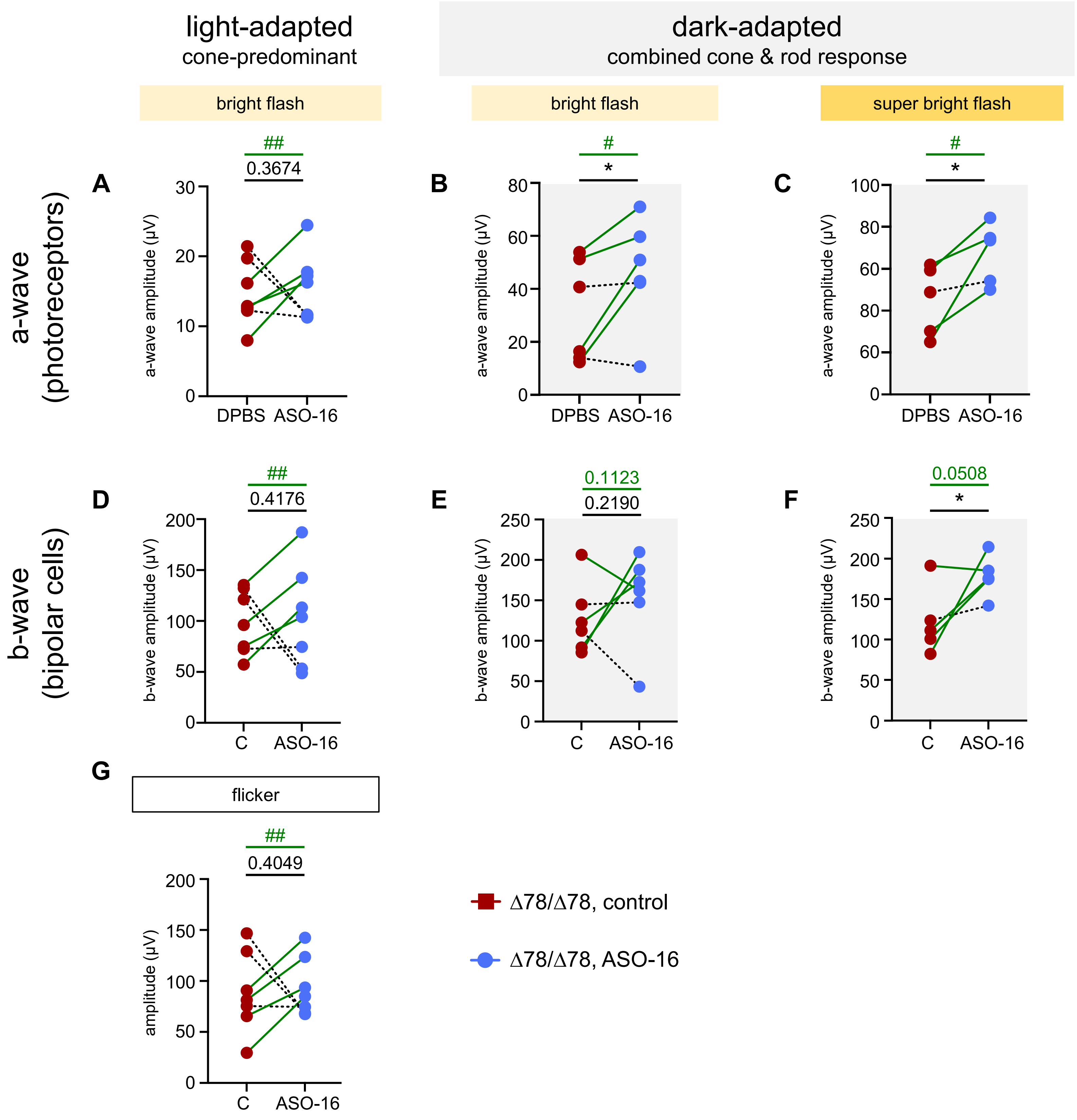

Figure S8. Intravitreal delivery of ASO-16 improves retinal function in a subset of high-responding pigs. (**A-G**) Comparison of the raw ERG amplitudes of individual *CLN3*$^{\Delta 78}$ pigs 9 months post-treatment (**A-C**) a-wave, (**D-F**) b-wave, and (**G**) amplitudes at 28.3 Hz flicker. Comparing vehicle-treated and 80 µg ASO-16 treated eyes within each *CLN3*$^{\Delta 78}$ pig with bright flash (8.0 cd•s/m^2) or super bright flash (25.0 cd•s/m^2). Connecting lines indicate which data points correspond to contralateral eyes within the same animal, with solid green lines, highlighting high-responding animals. One-tailed paired t-test comparisons for all animals shown in black; *P <0.05; one-tailed paired t-test comparisons for high-responding animals shown above in green lettering; $^{\#}$P<0.05, $^{\#\#}$P<0.01.

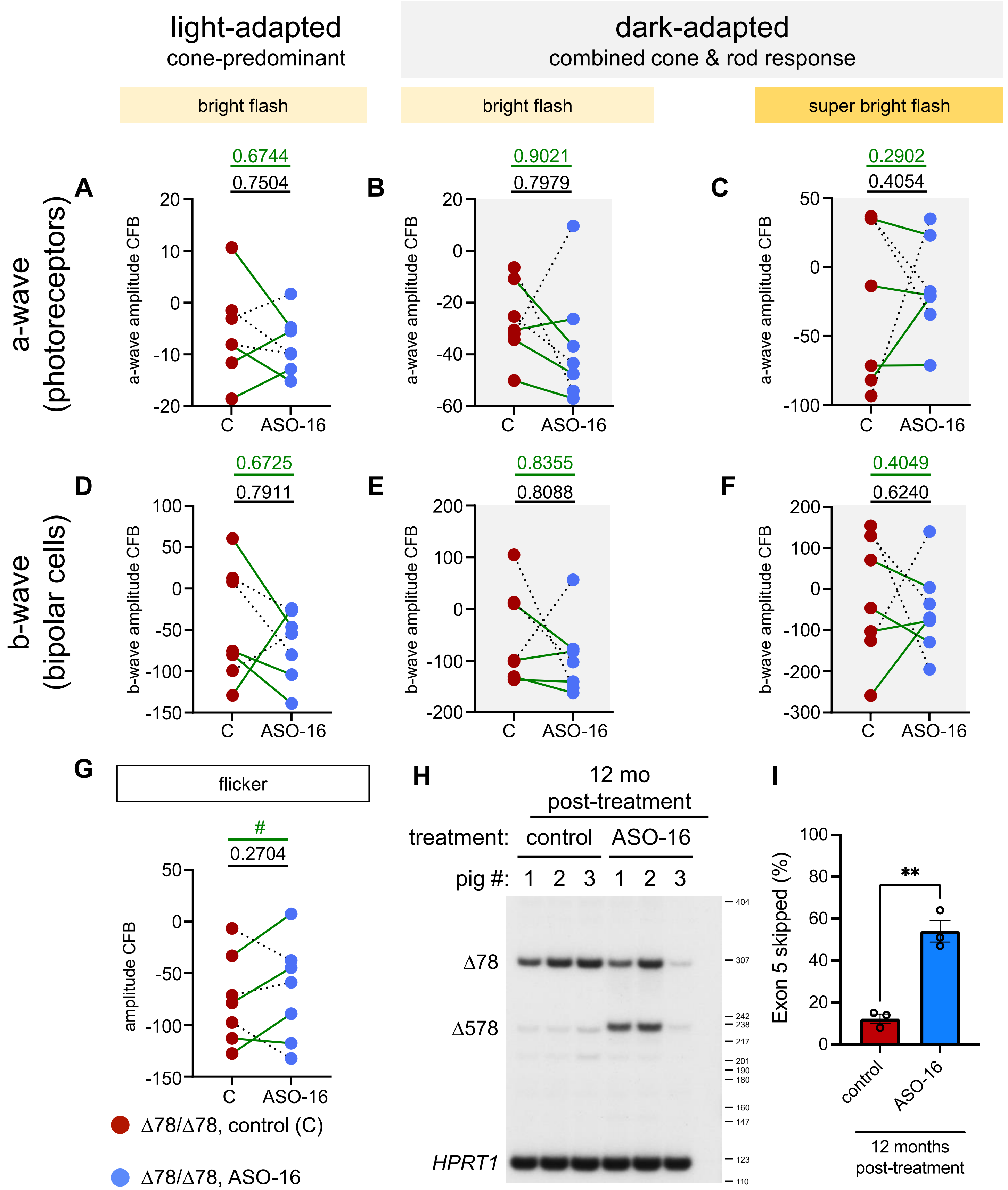

Figure S9. Retinal function and splicing 12 months after a single treatment with ASO-16. (**A-G**) The change from baseline (CFB) in ERG amplitudes of individual *CLN3*^{Δ78} pigs 12 months post-treatment (**A-C**) a-wave (predominately photoreceptors), (**D-F**) b-wave (predominately bipolar cells), and (**G**) amplitudes at 28.3 Hz flicker (cone bipolar cells only). Comparing vehicle-treated and 80 µg ASO-16 treated eyes within each *CLN3*^{Δ78} pig with bright flash (8.0 cd•s/m^2) or super bright flash (25.0 cd•s/m^2). Connecting lines indicate which data points correspond to contralateral eyes within the same animal, with solid green lines highlighting high-responding animals from 9 months post-treatment. One-tailed paired t-test comparisons for all animals shown in black; one-tailed paired t-test comparisons for high-responding animals shown in green; $^{\#}$P<0.05. (**H**) Products of radioactive RT–PCR analysis of pig retinal RNA isolated from three homozygous *CLN3*^{Δ78} pigs a year after intravitreal injection of DPBS vehicle (control) into one eye and ASO-16 (80 µg) into the other eye separated by PAGE. *HPRT1* was included as a loading control. Products are labeled on the left of the gel. Size markers (bp) are shown on the right of gel. (**I**) Quantification of exon 5 splicing (calculated as a percentage *CLN3*^{Δ578}/(*CLN3*^{Δ78}+*CLN3*^{Δ578})x100. Bars show s.e.m; paired t-test. **P <0.01.

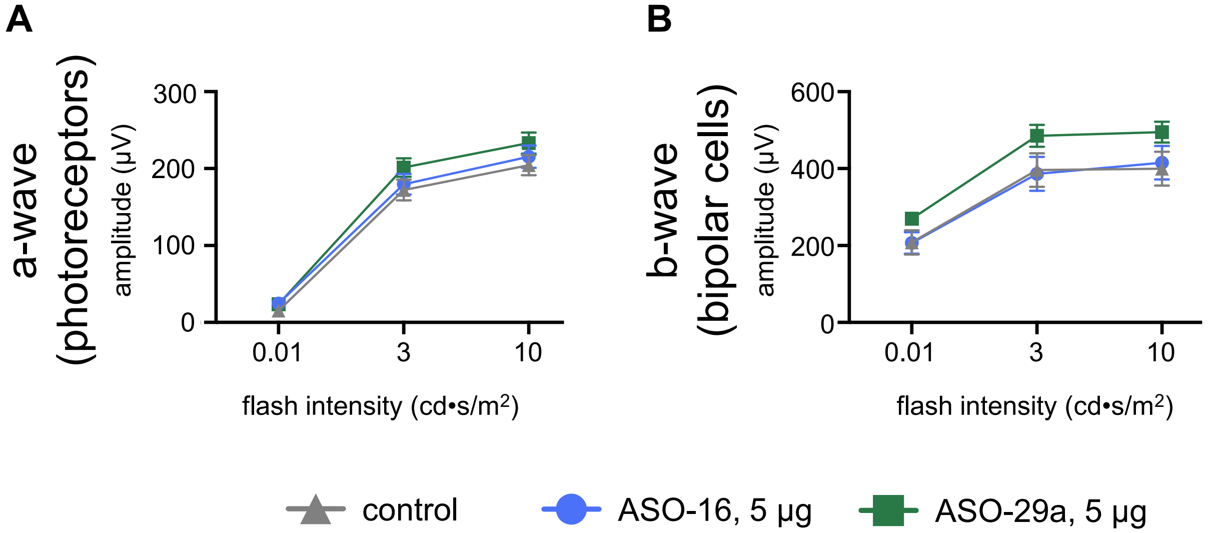

Figure S10. A single intravitreal injection of ASO-16 and ASO-29a is well tolerated in rats. Dark-adapted ERG of the (**A**) a-wave amplitude and (**B**) b-wave amplitude comparing the ASO-treated and vehicle-treated eye of wildtype rats 4 weeks post-treatment after the indicated form of stimulation. Bars show s.e.m.; one-way ANOVA comparing ASO-16 and ASO-29a to control treated.

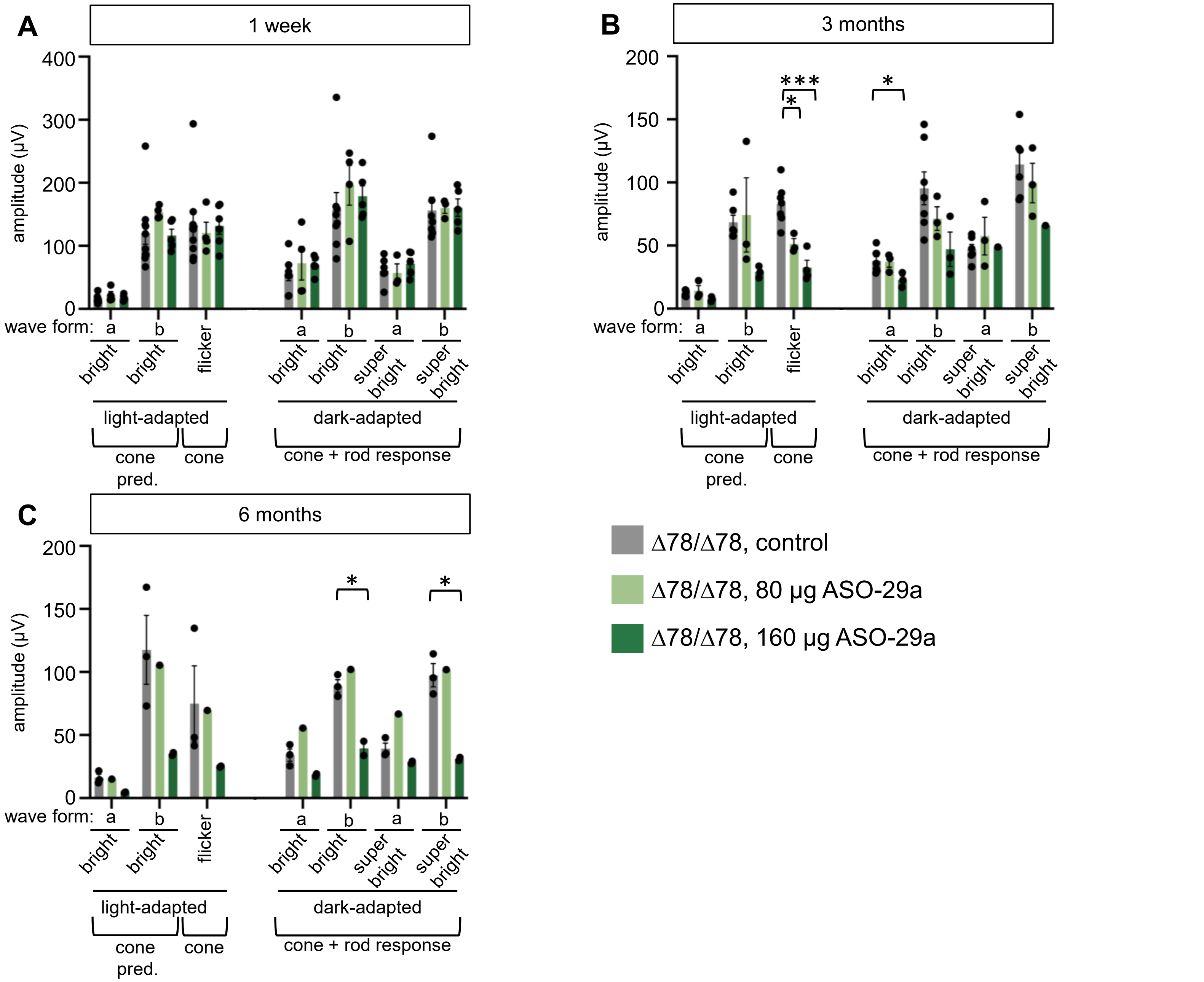

Figure S11. An 80 µg dose of ASO-29a is well-tolerated in the retina of *CLN3*$^{\Delta 78}$ minipigs. ERG raw amplitudes of a-wave, b-wave, and 28.3 Hz flicker following bright (8.0 cd•s/m^2) and super bright (25.0 cd•s/m^2) light stimuli in a light adapted or dark-adapted environment at (**A**) 1 week, (**B**) 3 months, and (**C**) 6 months post injection of DPBS or with either 80 µg or 160 µg of ASO-29a. Bars show s.e.m.; one-way ANOVA with Dunnett's multiple comparisons of vehicle treated to 80 µg and 160 µg ASO-29a treated; *P <0.05, ***P <0.001.

Summary of ocular AEs

Figure 2

	0.9% saline (n = 6)	DPBS (n = 3)	ASO-16, 300 µg (n = 2)	ASO-16, 200 µg (n = 4)	ASO-16, 80 µg (n = 3)	Relationship to study drug
Age at treatment (days)	11-30	125-154	11-30	11-30	125-154	
Number of eyes (%)						
**Ocular AE**	**1 (16.7)**	**1 (33.3)**	**2 (100)**	**3 (75)**	**0 (0)**	
cataract	1 (16.7)	0	0	0	0	not related
cloudy vitreous	1 (16.7)	0	0	0	0	not related
corneal ulcer	0	1 (33.3)	0	0	0	not related
diminished ERG	0	0	2 (100)	3 (75)	0	probable
**Non-ocular AE**	**0 (0)**	**0 (0)**	**0 (0)**	**4 (100)**	**0 (0)**	
(possible) influenza	0	0	0	3 (75)	0	unlikely
GI issues resulting in humane endpoint due to weight loss	0	0	0	1 (25)	0	unlikely

Figure 3; S4; S5

	DPBS (n = 15)	ASO-16, 80 µg (n = 9)	Relationship to study drug
Age at treatment (days)	103-154	111-154	
Number of eyes (%)			
**Ocular AE**	**1 (6.7)**	**0 (0)**	
cataract	1 (6.7)	0	not related
**Non-ocular AE**	**2 (13.3)**	**1 (11.1)**	
(possible) meningitis	1 (6.7)	0	unlikely
leg injury	1 (6.7)	1 (11.1)	unlikely

Figure 6; S11

	DPBS (n = 5)	ASO-25, 80 µg (n = 5)	ASO-25, 160 µg (n = 5)	Relationship to study drug
Age at treatment (days)	103-178	103-178	103-118	
Number of eyes (%)				
**Ocular AE**	**1 (20)**	**0 (0)**	**1 (20)**	
cataract	1 (20)	0	1 (20)	possible

Table S1. Ocular and Non-ocular Adverse Events.

Fig 3D: light-adapted, 8.0 cd•s/m² bright flash		p-value (sample number, N)		
a-wave CFB		3 mo	6 mo	9 mo
paired t-test	Δ78/Δ78, C vs. Δ78/Δ78, ASO-16 (80 µg)	0.0570 (7)	0.3416 (7)	0.2877 (7)
one-way ANOVA	+/Δ78, C vs. Δ78/Δ78, C	**0.0090** (6, 7)	0.1566 (6, 7)	0.0748 (6, 7)
	+/Δ78, C vs. Δ78/Δ78, ASO-16 (80 µg)	0.1357 (6, 7)	0.2549 (6, 7)	0.1982 (6, 7)

Fig 3E: dark-adapted, 8.0 cd•s/m² bright flash		p-value		
a-wave CFB		3 mo	6 mo	9 mo
paired t-test	Δ78/Δ78, C vs. Δ78/Δ78, ASO-16 (80 µg)	0.6664 (6)	0.9592 (6)	0.0965 (6)
one-way ANOVA	+/Δ78, C vs. Δ78/Δ78, C	0.4134 (6, 6)	0.1665 (6, 7)	0.2150 (6, 7)
	+/Δ78, C vs. Δ78/Δ78, ASO-16 (80 µg)	0.1858 (6, 6)	0.0028 (6, 6)	0.7254 (6, 6)

Fig 3F: dark-adapted, 25.0 cd•s/m² bright flash		p-value		
a-wave CFB		3 mo	6 mo	9 mo
paired t-test	Δ78/Δ78, C vs. Δ78/Δ78, ASO-16 (80 µg)	0.2376 (7)	0.3376 (5)	**0.0025** (5)
one-way ANOVA	+/Δ78, C vs. Δ78/Δ78, C	0.8493 (6, 7)	0.6126 (6, 7)	0.1809 (6, 6)
	+/Δ78, C vs. Δ78/Δ78, ASO-16 (80 µg)	0.9961 (6, 7)	0.9604 (6, 5)	0.7098 (6, 5)

Fig 3G: light-adapted, 8.0 cd•s/m² bright flash		p-value		
b-wave CFB		3 mo	6 mo	9 mo
paired t-test	Δ78/Δ78, C vs. Δ78/Δ78, ASO-16 (80 µg)	**0.0062** (7)	**0.0074** (7)	**0.0497** (7)
one-way ANOVA	+/Δ78, C vs. Δ78/Δ78, C	**0.0251** (6, 7)	0.7795 (6, 7)	**0.0359** (6, 7)
	+/Δ78, C vs. Δ78/Δ78, ASO-16 (80 µg)	0.6512 (6, 7)	0.6435 (6, 7)	0.8037 (6, 7)

Fig 3H: dark-adapted, 8.0 cd•s/m² bright flash		p-value		
b-wave CFB		3 mo	6 mo	9 mo
paired t-test	Δ78/Δ78, C vs. Δ78/Δ78, ASO-16 (80 µg)	0.3827 (6)	0.7515 (6)	0.2182 (6)
one-way ANOVA	+/Δ78, C vs. Δ78/Δ78, C	0.1107 (6, 6)	**0.0023** (6, 7)	**0.0367** (6, 7)
	+/Δ78, C vs. Δ78/Δ78, ASO-16 (80 µg)	0.2114 (6, 6)	**0.0006** (6, 6)	0.2022 (6, 6)

Fig 3I: dark-adapted, 25.0 cd•s/m² bright flash		p-value		
b-wave CFB		3 mo	6 mo	9 mo
paired t-test	Δ78/Δ78, C vs. Δ78/Δ78, ASO-16 (80 µg)	0.1892 (7)	0.2696 (5)	**<0.0001** (5)
one-way ANOVA	+/Δ78, C vs. Δ78/Δ78, C	0.6045 (6, 7)	0.1700 (6, 7)	0.1488 (6, 6)
	+/Δ78, C vs. Δ78/Δ78, ASO-16 (80 µg)	0.9940 (6, 7)	0.4226 (6, 5)	0.8304 (6, 5)

Fig 3K: 8.0 cd•s/m² flicker at 28.3 Hz		p-value		
amplitude CFB		3 mo	6 mo	9 mo
paired t-test	Δ78/Δ78, C vs. Δ78/Δ78, ASO-16 (80 µg)	**0.0183** (7)	0.1218 (7)	0.0898 (7)
one-way ANOVA	+/Δ78, C vs. Δ78/Δ78, C	**0.0032** (6, 7)	0.1443 (6, 7)	**0.0218** (5, 7)
	+/Δ78, C vs. Δ78/Δ78, ASO-16 (80 µg)	0.6268 (6, 7)	0.7008 (6, 7)	0.4200 (5, 7)

Table S2. ERG p-values corresponding to results from Figure 3. Sample size (N) is indicated in parenthesis next to p-value for each comparison. In some cases, a measurement is missing due to technical issues as described in methods.

Fig 4A: light-adapted, 8.0 cd•s/m^2 bright flash		p-value (sample number, N)
a-wave CFB		9 mo
paired t-test	Δ78/Δ78: C vs. ASO-16 (80 µg), high responders	0.0958 (4)
paired t-test	Δ78/Δ78: C vs. ASO-16 (80 µg), all animals	0.2877 (7)

Fig 4B: dark-adapted, 8.0 cd•s/m^2 bright flash		p-value
a-wave CFB		9 mo
paired t-test	Δ78/Δ78: C vs. ASO-16 (80 µg), high responders	0.0322 (4)
paired t-test	Δ78/Δ78: C vs. ASO-16 (80 µg), all animals	0.0965 (6)

Fig 4C: dark-adapted, 25.0 cd•s/m^2 bright flash		p-value
a-wave CFB		9 mo
paired t-test	Δ78/Δ78: C vs. ASO-16 (80 µg), high responders	**0.0049** (4)
paired t-test	Δ78/Δ78: C vs. ASO-16 (80 µg), all animals	**0.0025** (5)

Fig 4D: light-adapted, 8.0 cd•s/m^2 bright flash		p-value
b-wave CFB		9 mo
paired t-test	Δ78/Δ78: C vs. ASO-16 (80 µg), high responders	**0.0068** (4)
paired t-test	Δ78/Δ78: C vs. ASO-16 (80 µg), all animals	**0.0497** (7)

Fig 4E: dark-adapted, 8.0 cd•s/m^2 bright flash		p-value
b-wave CFB		9 mo
paired t-test	Δ78/Δ78: C vs. ASO-16 (80 µg), high responders	0.1120 (4)
paired t-test	Δ78/Δ78: C vs. ASO-16 (80 µg), all animals	0.2182 (6)

Fig 4F: dark-adapted, 25.0 cd•s/m^2 bright flash		p-value
b-wave CFB		9 mo
paired t-test	Δ78/Δ78: C vs. ASO-16 (80 µg), high responders	**0.0009** (4)
paired t-test	Δ78/Δ78: C vs. ASO-16 (80 µg), all animals	**<0.0001** (5)

Fig 4G: 8.0 cd•s/m^2 flicker at 28.3 Hz		p-value
amplitude CFB		9 mo
paired t-test	Δ78/Δ78: C vs. ASO-16 (80 µg), high responders	**0.0106** (4)
paired t-test	Δ78/Δ78: C vs. ASO-16 (80 µg), all animals	0.0898 (7)

Table S3. ERG p-values corresponding to results from Figure 4. Sample size (N) is indicated in parenthesis next to p-value for each comparison. In some cases, a measurement is missing due to technical issues as described in methods.

Fig S8A: light-adapted, 8.0 cd•s/m^2 bright flash		p-value (sample number, N)
a-wave CFB		9 mo
paired t-test	Δ78/Δ78: C vs. ASO-16 (80 µg), high responders	**0.0086** (4)
paired t-test	Δ78/Δ78: C vs. ASO-16 (80 µg), all animals	0.3674 (7)

Fig S8B: dark-adapted, 8.0 cd•s/m^2 bright flash		p-value
a-wave CFB		9 mo
paired t-test	Δ78/Δ78: C vs. ASO-16 (80 µg), high responders	**0.0166** (4)
paired t-test	Δ78/Δ78: C vs. ASO-16 (80 µg), all animals	**0.0325** (6)

Fig S8C: dark-adapted, 25.0 cd•s/m^2 bright flash		p-value
a-wave CFB		9 mo
paired t-test	Δ78/Δ78: C vs. ASO-16 (80 µg), high responders	**0.0209** (4)
paired t-test	Δ78/Δ78: C vs. ASO-16 (80 µg), all animals	**0.0192** (5)

Fig S8D: light-adapted, 8.0 cd•s/m^2 bright flash		p-value
b-wave CFB		9 mo
paired t-test	Δ78/Δ78: C vs. ASO-16 (80 µg), high responders	**0.0023** (4)
paired t-test	Δ78/Δ78: C vs. ASO-16 (80 µg), all animals	0.4176 (7)

Fig S8E: dark-adapted, 8.0 cd•s/m^2 bright flash		p-value
b-wave CFB		9 mo
paired t-test	Δ78/Δ78: C vs. ASO-16 (80 µg), high responders	0.1123 (4)
paired t-test	Δ78/Δ78: C vs. ASO-16 (80 µg), all animals	0.2190 (6)

Fig S8F: dark-adapted, 25.0 cd•s/m^2 bright flash		p-value
b-wave CFB		9 mo
paired t-test	Δ78/Δ78: C vs. ASO-16 (80 µg), high responders	0.0508 (4)
paired t-test	Δ78/Δ78: C vs. ASO-16 (80 µg), all animals	**0.0386** (5)

Fig S8G: 8.0 cd•s/m^2 flicker at 28.3 Hz		p-value
amplitude CFB		9 mo
paired t-test	Δ78/Δ78: C vs. ASO-16 (80 µg), high responders	**0.0027** (4)
paired t-test	Δ78/Δ78: C vs. ASO-16 (80 µg), all animals	0.4049 (7)

Table S4. ERG p-values corresponding to results from Figure S8. Sample size (N) is indicated in parenthesis next to p-value for each comparison. In some cases, a measurement is missing due to technical issues as described in methods.

Fig S9A: light-adapted, 8.0 cd•s/m^2 bright flash		p-value
a-wave CFB		12 mo
paired t-test	DPBS Δ78/Δ78 vs. ASO-16, 80 µg Δ78/Δ78, high responders	0.6744 (4)
paired t-test	DPBS Δ78/Δ78 vs. ASO-16, 80 µg Δ78/Δ78, all animals	0.7504 (7)

Fig S9B: dark-adapted, 8.0 cd•s/m^2 bright flash		p-value
a-wave CFB		12 mo
paired t-test	DPBS Δ78/Δ78 vs. ASO-16, 80 µg Δ78/Δ78, high responders	0.9021 (4)
paired t-test	DPBS Δ78/Δ78 vs. ASO-16, 80 µg Δ78/Δ78, all animals	0.7979 (7)

Fig S9C: dark-adapted, 25.0 cd•s/m^2 bright flash		p-value
a-wave CFB		12 mo
paired t-test	DPBS Δ78/Δ78 vs. ASO-16, 80 µg Δ78/Δ78, high responders	0.2902 (4)
paired t-test	DPBS Δ78/Δ78 vs. ASO-16, 80 µg Δ78/Δ78, all animals	0.4054 (7)

Fig S9D: light-adapted, 8.0 cd•s/m^2 bright flash		p-value
b-wave CFB		12 mo
paired t-test	DPBS Δ78/Δ78 vs. ASO-16, 80 µg Δ78/Δ78, high responders	0.6725 (4)
paired t-test	DPBS Δ78/Δ78 vs. ASO-16, 80 µg Δ78/Δ78, all animals	0.7911 (7)

Fig S9E: dark-adapted, 8.0 cd•s/m^2 bright flash		p-value
b-wave CFB		12 mo
paired t-test	DPBS Δ78/Δ78 vs. ASO-16, 80 µg Δ78/Δ78, high responders	0.8355 (4)
paired t-test	DPBS Δ78/Δ78 vs. ASO-16, 80 µg Δ78/Δ78, all animals	0.8088 (7)

Fig S9F: dark-adapted, 25.0 cd•s/m^2 bright flash		p-value
b-wave CFB		12 mo
paired t-test	DPBS Δ78/Δ78 vs. ASO-16, 80 µg Δ78/Δ78, high responders	0.4049 (4)
paired t-test	DPBS Δ78/Δ78 vs. ASO-16, 80 µg Δ78/Δ78, all animals	0.6240 (7)

Fig S9G: 8.0 cd•s/m^2 flicker at 28.3 Hz		p-value
amplitude		12 mo
paired t-test	DPBS Δ78/Δ78 vs. ASO-16, 80 µg Δ78/Δ78, high responders	**0.0423** (4)
paired t-test	DPBS Δ78/Δ78 vs. ASO-16, 80 µg Δ78/Δ78, all animals	0.2704 (7)

Table S5. ERG p-values corresponding to results from Figure S9. Sample size (N) is indicated in parenthesis next to p-value for each comparison.

ASOs	Sequence (5'-3')
1	CAGAGAACACAGTGAGAC
2	GGGACCAGAGAACACAGT
3	CGCCTGGGACCAGAGAAC
4	AGCACCGCCTGGGACCAG
5	CCAGGAGCACCGCCTGGG
6	GTCTGCCAGGAGCACCGC
7	AGGATGTCTGCCAGGAGC
8	TGGGAAGGATGTCTGCCA
9	GAGGGTGGGAAGGATGTC
10	ATGATGAGGGTGGGAAGG
11	ATTTGATGATGAGGGTGG
12	CAGCAATTTGATGATGAG
13	GGAGCCAGCAATTTGATG
14	CAAGAGGAGCCAGCAATT
15	GAGGCCAAGAGGAGCCAG
16	AGATGGAGGCCAAGAGGA
17	GCAGCAGATGGAGGCCAA
18	GTAGGGCAGCAGATGGAG
19	GACCTGTAGGGCAGCAGA
20	CCCCAGACCTGTAGGGCA
21	CCCCTCCCCAGACCTGTA
22	CCCACCCCCTCCCCAGAC
23	CCACCCCCACCCCCTCCC
24	TCCCACCCCCACCCCCTC
29a	CCCAGACCTGTAGGGCAG
29b	CCAGACCTGTAGGGCAGC
29c	CAGACCTGTAGGGCAGCA
29d	AGACCTGTAGGGCAGCAG
**Primers**	**Sequence (5'-3')**
pCLN3ex3F	AGTGCTGCCCACGACATC
pCLN3ex4F	TAACTCTGTCTCCACGGC
pCLN3 ex6R	TTCCAGCGGCACAGATCC
pCLN3in6F	TGGTTTGCTAACTGGGTGGG
pCLN3in8R	TCACCACATGGAGAAGGAAGC
pCLN3ex10R	ACAGGTGTGAGCCGGAGC
pHPRT1F	TTATGGACAGGACTGAACGGC
pHPRT1R	GTAATCCAGCAGGTCAGCAAAG
hCLN3 ex4	GCAACTCTGTCTCTACGGC
hCLN3 ex10	CTTGAACACTGTCCACC

Table S6. Splice switching antisense oligonucleotide and primer sequences.

A **Genomic matches of ASO-29a subsequences**

Length (nt)	Number of possible subsequences	Number of off-target hits	Off-target locus (hg19)	Gene annotation (RefSeq)		
				Gene	Strand	Region
18	1	0	-	-	-	-
17	2	0	-	-	-	-
16	3	6	chr1:970996-971013	*AGRN*	sense	intron (291 bp away from junction)
			chr6:1859273-1859290	*GMDS*	antisense	intron (>71kb away from junction)
			chr11:117908797-117908814	*SMIM35*	sense	intron (>6kb away from junction)
			chr13:55752072-55752089	-	-	-
			chr15:86917573-86917590	*AGBL1*	antisense	intron (>23kb away from junction)
			chr17:72822712-72822729	*TMEM104*	antisense	intron (>6.7kb away from junction)

B **Genomic matches of nusinersen subsequences**

Length (nt)	Number of possible subsequences	Number of off-target hits	Off-target locus (hg19)	Gene annotation (RefSeq)		
				Gene	Strand	Region
18	1	0	-	-	-	-
17	2	3	chr2: 151151798-151151814	-	-	-
			chr6: 137801026-137801042	-	-	-
			chr12: 129939692-129939708	*TMEM132D*	sense	intron (>75 kb away from junction)
16	3	7	chr13: 30033807-30033822	*MTUS2*	antisense	intron (>19kb away from junction)
			chr3: 82606750-82606765	-	-	-
			chr15: 32439151-32439166	*CHRNA7*	antisense	intron (>6.9kb away from junction)
			chr20: 9638297-9638312	*PAK5*	sense	intron (>13kb away from junction)
			chr7: 125728305-125728320	-	-	-
			chr7: 141917297-141917312	*MGAM2*	antisense	intron (>800bp away from junction)
			chr3: 110103460-110103475	-	-	-

Table S7. Genomic off-target footprints of ASO-29a, compared to those of nusinersen. (**A**) All possible coding subsequences of a given length were taken from the ASO-29a sequence and aligned to the reference human genome in the *GRCh37/hg19* database. The number and identity of the off-target matches are shown. If the off-target locus is in an intron, the distance from the closest splice junction is indicated. (**B**) The same alignment was performed as in panel A, but for nusinersen in the human genome.

Figure 2																
ASO Dose	vehicle		300 µg		vehicle			200 µg			vehicle			80 µg		
Time post-IVI	3 mo	6 mo	3 mo	6 mo	3 mo	6 mo	12 mo	3 mo	6 mo	12 mo	3 mo	6 mo	12 mo	3 mo	6 mo	12 mo
Sample Size (# of eyes)	1	1	1	1	1	1	1	1	1	1	1	1	1	1	1	1

Figure 3, S4, S5			
Time post-IVI	DPBS +/Δ78	DPBS Δ78/Δ78	ASO-16, 80 µg Δ78/Δ78
1 week	7 (5M, 2F)	9 (6M, 3F)	9 (6M, 3F)
3 months	7 (5M, 2F)	9 (6M, 3F)	9 (6M, 3F)
6 months	7 (5M, 2F)	9 (6M, 3F)	9 (6M, 3F)
9 months	7 (5M, 2F)	9 (6M, 3F)	9 (6M, 3F)

Figure 6												
ASO Dose	vehicle			80 µg			vehicle			160 µg		
Time post-IVI	1 mo	3 mo	6 mo	1 mo	3 mo	6 mo	1 mo	3 mo	6 mo	1 mo	3 mo	6 mo
Sample Size (# of eyes)	2	2	1	2	2	1	1	2	2	1	2	2

Figure S3								
Genotype	WT				Δ78			
Age	2	6	36	50	2	6	36	50
Sample Size	2	1	1	1	2	2	3	2

Figure S10			
Treatment	DPBS	ASO-16	ASO-29a
Genotype	WT	WT	WT
Sample Size (# of eyes)	4	8	8

Figure S11			
Time post-IVI	DPBS Δ78/Δ78	ASO-29a, 80 µg Δ78/Δ78	ASO-29a, 160 µg Δ78/Δ78
1 week	10 (4M, 6F)	4 (2M, 2F)	5 (2M, 3F)
3 months	6 (4M, 2F)	3 (3M, 0F)	3 (2M, 1F)
6 months	3 (2M, 1F)	1 (1M, 0F)	2 (1M, 1F)

Table S8. Experimental Sample Sizes.
